# Enhancement of Th1/Th17 inflammation by TRIM21 in Behçet’s disease

**DOI:** 10.1038/s41598-017-03251-5

**Published:** 2017-06-07

**Authors:** Yuri Ahn, Ji-Hye Hwang, Zhenlong Zheng, Dongsik Bang, Do-Young Kim

**Affiliations:** 1 Department of Dermatology and Cutaneous Biology Research Institute, Yonsei University College of Medicine, Seoul, Korea; 2 Department of Dermatology, Yanbian University Hospital, Yanji, China; 3 Department of Dermatology, Catholic Kwandong University, International St. Mary’s Hospital, Incheon, Korea

## Abstract

The etiology of Behçet’s disease (BD), a chronic, multisystemic autoinflammatory and autoimmune disease, remains unknown; however, researchers have postulated that infectious agents, such as herpes simplex virus, are significant triggering factors of BD. Tripartite motif-containing (TRIM) proteins exhibit antiviral properties, mediating antiviral defense mechanisms. The purpose of this study was to investigate TRIM21 protein expression in the monocytes of BD patients and to identify the role of TRIM21 in immune dysregulation in BD. In this study, the expression of TRIM21 and related molecules, including interferon regulatory factor 8 (IRF8), was analyzed in monocytes from BD patients. Functional analyses using small interfering RNA and co-culture with responder T cells were performed to examine the pathological role of TRIM21 in BD. Peripheral blood monocytes from BD patients showed increased TRIM21 expression and decreased IRF8 expression compared with that in monocytes from healthy controls. TRIM21 was found to decrease IRF8 expression. BD monocytes facilitated Th1 and Th17 differentiation of co-cultured T cells, and knock-down of TRIM21 expression by small interfering RNA inhibited this differentiation. In conclusion, TRIM21 played a pivotal role in regulating the secretion of proinflammatory cytokines in monocytes of BD patients.

## Introduction

The tripartite motif-containing (TRIM) family of proteins has been recognized for its role in regulating the innate immune response to viral infections. TRIM proteins are structurally characterized by a Really Interesting New Gene (RING) domain, a B-box domain, and a coiled-coil domain. TRIM21 is an E3 ubiquitin ligase that regulates innate immune responses by ubiquitinating various intracellular proteins, such as nuclear factor kappa B (NF-kB) and interferon (IFN) regulatory factors (IRFs). NF-κB is widely involved in the expression of proinflammatory genes, including cytokines, chemokines, and adhesion molecules, and TRIM21 promotes NF-kB signaling, especially during infectious conditions^1^. Multiple IRFs are tightly regulated by TRIM21 in immune cells^2^. For example, TRIM21 interacts and ubiquitinates IRF3, IRF7, and IRF8^3, 4^. IRFs play a critical role in inhibiting Th17-cell differentiation, acting as intrinsic transcriptional inhibitors^5^.

Behçet’s disease (BD) is a chronic, multisystemic, inflammatory condition characterized by mucocutaneous lesions and vasculitis on various organs^6^. Although the exact etiology remains unknown, dysregulated innate and acquired immune systems have been implicated in the pathogenesis^7^. Recent studies have shown that Th1 and Th17 T cells play the pivotal pathogenic role in BD^8–10^. However, the mechanism of these pathologic T-cell responses remains elusive. Because human monocytes trigger and polarize Th responses and regulate T-cell responses during infection and in autoimmune diseases, monocytes and their progeny, such as macrophages, appear to play an important role in BD pathogenesis^11–13^.

Based on the hypothesized role of monocytes in BD and previous observations of TRIM21 in other immune related disorders, we decided to investigate the functional role of monocyte TRIM21 in the abnormal T-cell response in BD. Thus, we sought to elucidate TRIM21 protein expression in monocytes of BD patient and to identify the role of TRIM21 on Th1/Th17 immune deviation in BD.

## Materials and Methods

### Ethics Statement

Each participant provided written informed consent before enrollment in this study, and the protocols was approved by the Institutional Review Board of Severance Hospital, Yonsei University College of Medicine, Seoul, Korea (IRB number: 4-2016-0391) in accordance with the ethical guidelines of the Helsinki Declaration. All samplings and experiments followed the approved guidelines.

### Patients

Korean BD patients who visited the BD Specialty Clinic of Severance Hospital and fulfilled the diagnostic criteria of the International Study Group for BD and healthy control volunteers were recruited^14^. Patients with other concomitant autoimmune diseases, malignancies, or infections were excluded from the study. The clinical features of BD were identified by trained dermatologists based on medical history and physical examination. Venous blood samples were collected from all participants.

### Isolation of monocytes and T cells from peripheral blood

Peripheral blood mononuclear cells (PBMCs) were separated from heparinized venous blood using Ficoll-Hypaque (GE Healthcare, Buckinghamshire, UK) density-gradient centrifugation. Highly purified T cells and monocytes were prepared from PBMCs by exhaustive immunomagnetic negative selection in an MACS separator (Miltenyi Biotec, Bergish Gladbach, Germany) using a T Cell Negative Isolation Kit and a Pan Monocyte Negative Isolation Kit (Miltenyi Biotec, Bergish Gladbach, Germany).

### Small interfering RNA(siRNA) transfection

Four different *TRIM21*-targeting siRNAs was purchased from Qiagen (Germany). Primary monocytes and THP-1 cells were transfected with 10 nM of siRNA using lipofectamine 2000 (Invitrogen, USA) according to the manufacturer’s protocols. Knock-down efficiencies were analyzed 24 h after transfection by western blot analysis.

### Confocal microscopy

Primary monocytes were grown for 24 h on Lab-Tek eight-well Permanox chamber slides (Thermo Fisher Scientific, San Jose, USA). Cells were fixed with 4% paraformaldehyde for 10 min and permeabilized with 0.1% Triton X-100. After blocking with 1% bovine serum albumin in phosphate-buffered saline for 30 min, they were then incubated with anti-TRIM21 Alexa 488 (Bioss, Woburn, USA) for 1 h at 37 °C in the dark. The cells were washed, and mounting media with 4′,6-diamidino-2-phenylindole (DAPI) was added to the slide. The slide was analyzed with a ZEISS immunoconfocal microscope.

### Western blot

Freshly isolated monocytes and stimulated monocytes were lysed in radioimmunoprecipitation assay buffer containing protease inhibitors and phosphatase inhibitor (GeneDipot, Barker, USA). Protein was quantified using bicinchoninic acid assay. Cell lysates were resolved by sodium dodecyl sulfate polyacrylamide gel electrophoresis and analyzed by western blot using specific antibodies against TRIM21 (Proteintech, USA), IRF8 (Cell signaling, UK), and phosphor-p65 (Cell signaling). Western blot band signal densities for TRIM21, IRF8, and phosphor-p65 were normalized to those of a loading control (glyceraldehyde 3-phosphate dehydrogenase [GAPDH]) and averaged from three independent experiments.

### Real-time polymerase chain reaction (RT-PCR)

For RNA isolation, cells were harvested and total RNA isolated using Trizol reagent (Invitrogen) according to the manufacturer’s protocol. cDNA was synthesized with an oligo (dT) primer and the Superscript II reverse transcriptase system followed by RT-PCR using SYBR Green Master Mix (Applied Biosystems, Foster City, USA). Primer sequences were as follows: interleukin(IL)-17A, forward ACCAATCCCAAAAGGTCCTC and reverse GGGGACAGAGTTCATGTGGT; IFN-γ, forward TGACCAGAGCATCCAAAAGA and reverse CTCTTCGACCTCGAAACAGC; IL-4, forward GCCACCATGAGAAGGACACT and reverse ACTCTGGTTGGCTTCCTTCA; IL-13, forward GTACTGTGCAGCCCTGGAAT and reverse TTTACAAACTGGGCCACCTC.

### NF-kB luciferase assay

THP-1 cells were seeded at a density of 65,000 cells per well in 12-well plates 1 day prior to transfection. Cells were transfected with NF-kB-Luc reporter plasmid (500 ng/ml) and β-galactosidase (β-gal, 500 ng/ml) for 24 h. Cells were harvested and luciferase assay performed using the Luciferase Assay Kit (Promega, USA) according to the manufacturer’s protocol. Cells were lysed in 150 μl 1 × lysis buffer and centrifuged at 12,000 g for 15 s, and luminescence in the supernatant was measured using the luciferase assay reagent. Values were normalized by results of a β-gal assay using the β-galactosidase Kit (Promega). For this assay, 50 μl of the lysate supernatant was incubated with 50 μl 2 × assay buffer at 37 °C for 30 min, and the resulting yellow color was quantified by absorbance at 420 nm. β-gal activity in each of the lysates was obtained using a standard curve, and NF-kB-luciferase activity was normalized against β-gal activity, averaged for three independent sets of experiments, and plotted.

### Co-culture conditions

Monocytes (4 × 10^5^/ml) and T cells (4 × 10^5^/ml) were seeded in 12-well plastic tissue culture plates (Corning, Cambridge, USA). Co-cultures were maintained for 7 days in complete Roswell Park Memorial Institute medium 1640 (Hyclone) containing 10% fetal bovine serum, L-glutamine (2 mM), penicillin (100 units/ml), and streptomycin (100 μg/ml) in the presence of 1 μg/ml soluble anti-CD3 antibody (eBioscience, USA) and 100 ng/ml lipopolysaccharide (LPS, Sigma, USA). After 7 days of culture, cells were stimulated with 50 ng/ml phorbol myristate acetate (PMA; Sigma) and 1 μg/ml ionomycin (Sigma) for 6 h, with Golgistop (BD Biosciences, USA) present for the last 4 h. For intracellular staining, we used a human Th1/Th17 phenotype kit for flow cytometry according to the manufacturer’s protocol (BD Biosciences).

### Flow cytometry

For intracellular staining, monocytes from healthy and BD patients were incubated with anti-human TRIM21 for 30 min at RT and added to FITC-conjugated anti-IgG secondary antibody for 15 min at RT. HeLa cells, which have elevated TRIM21 levels, also used as positive control. For co-cultured CD4^+^ cells, we used a human Th1/Th17 phenotype kit for flow cytometry according to the manufacturer’s protocol (BD Biosciences). Cells were stained for 30 min at RT with the following antibody cocktail: FITC-conjugated anti-human IFN-γ, PE-conjugated anti-human IL-17A, and PerCP-Cy5.5-conjugated anti-human CD4. After washing, cells were fixed in 4% paraformaldehyde and analyzed using an LSRII cytometer (BD Biosciences) and Flowjo software.

### Enzyme-linked immunosorbent assay (ELISA)

Supernatants from the co-cultures were collected, centrifuged to remove cell debris, and stored at −70 °C for future cytokine measurements. The IL-17 levels (eBioscience) were determined using an ELISA kit according to the manufacturer’s instructions. Supernatants from healthy and BD monocytes after LPS stimulation were analyzed with IL-6, IL-1β, IL-23 (eBioscience), and IL-12/23p40 (R&D Systems, USA) ELISA kits according to the manufacturers’ instructions.

### Statistical analyses

Statistical analyses were conducted using Graph Pad Prism, version 4.03 (GraphPad Software, Inc., San Diego, CA). Data are expressed as the mean ± standard deviation (SD) for parametric data and as the median and interquartile range for nonparametric data. The significance of differences between two groups was determined using a two-tailed Student’s t-test or Wilcoxon signed-rank test. For multiple comparisons, one-way analysis of variance with a Bonferroni test was used. A P-value < 0.05 was considered statistically significant.

## Results

### Increased TRIM21 expression in BD monocytes

**Figure 1:**
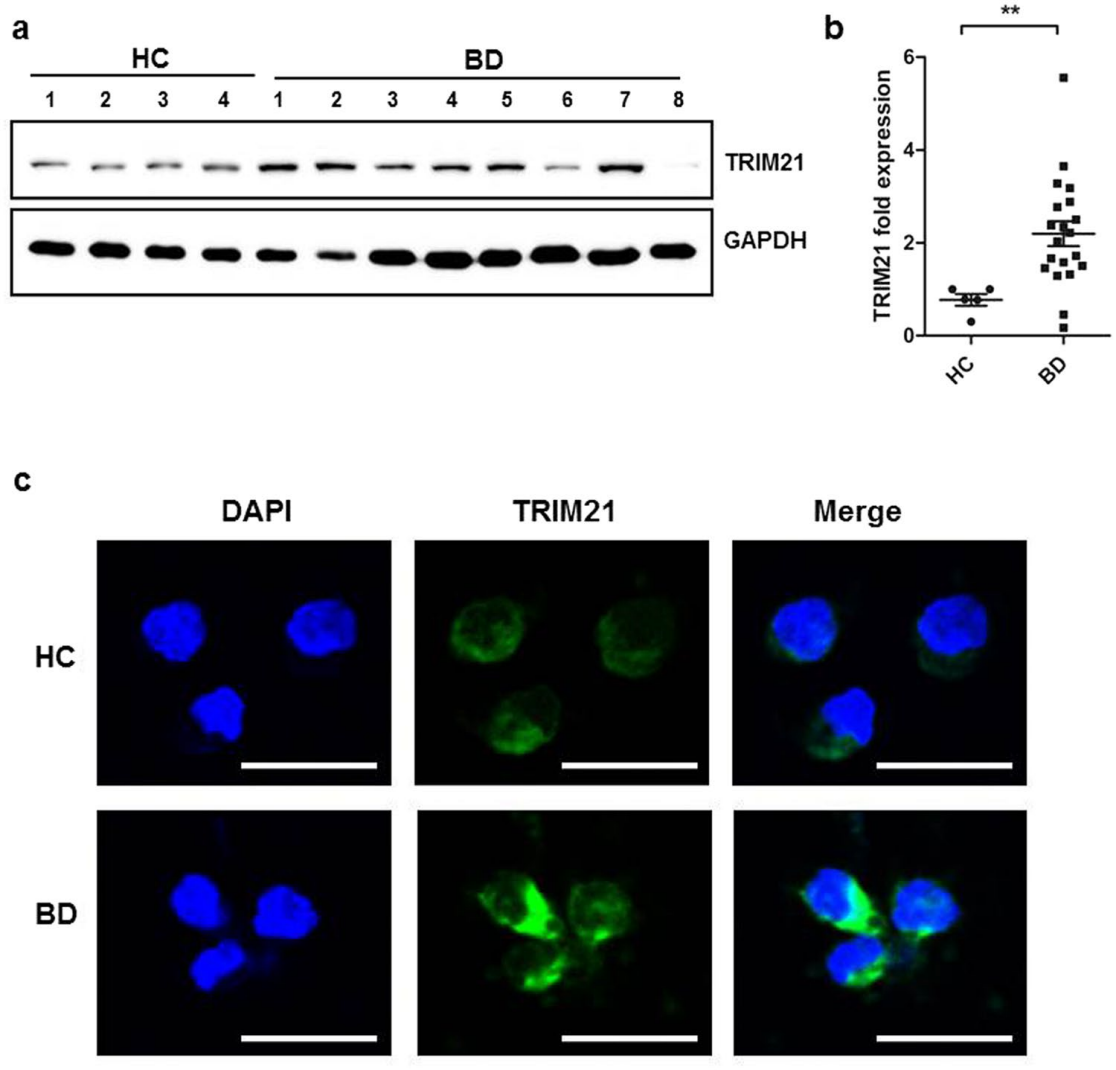
Monocytes from Behçet’s disease patients exhibited increased TRIM21 expression compared to those from healthy controls. (**a** and **b**) Human monocytes isolated from healthy volunteers and BD patients. TRIM21 protein levels in monocytes from 18 BD patients and five healthy control subjects, as determined by western blot (**a**). TRIM21 expression was quantified by densitometry following normalization to GAPDH expression as indicated in accompanying graph (**b**). Data are expressed as the mean ± SD. **P < 0.01. The cropped blots are displayed and full-length blots are shown in the Supplementary Information. (**c**) Endogenous TRIM21 was visualized by immunofluorescence using anti-TRIM21 (green) and DAPI (blue) and observed under confocal microscopy. Scale bar represents 20 μm.

To confirm the clinical significance of TRIM21 in BD, we first examined the expression of TRIM21 in freshly isolated monocytes from BD patients and healthy controls. Western blot analysis showed that TRIM21 expression in monocytes was significantly higher in BD patients (n = 18) than in healthy controls (n = 5) (P = 0.0009, Fig. 1a and b). Confocal immunofluorescence microscopy using an anti-TRIM21 antibody also revealed greater TRIM21 expression in both the cytosol and nucleus of monocytes from BD patients compared with healthy controls (Fig. 1c). TRIM21 expression in BD monocytes was also validated using flow cytometric analysis (Supplementary Figure 1A). HeLa cells, which have elevated TRIM21 levels, were used as positive control^15^. From these observations, we confirmed that TRIM21 expression was significantly higher in monocytes from BD patients.

### IRF8, a representative downstream target of TRIM21, was decreased in BD monocytes

**Figure 2:**
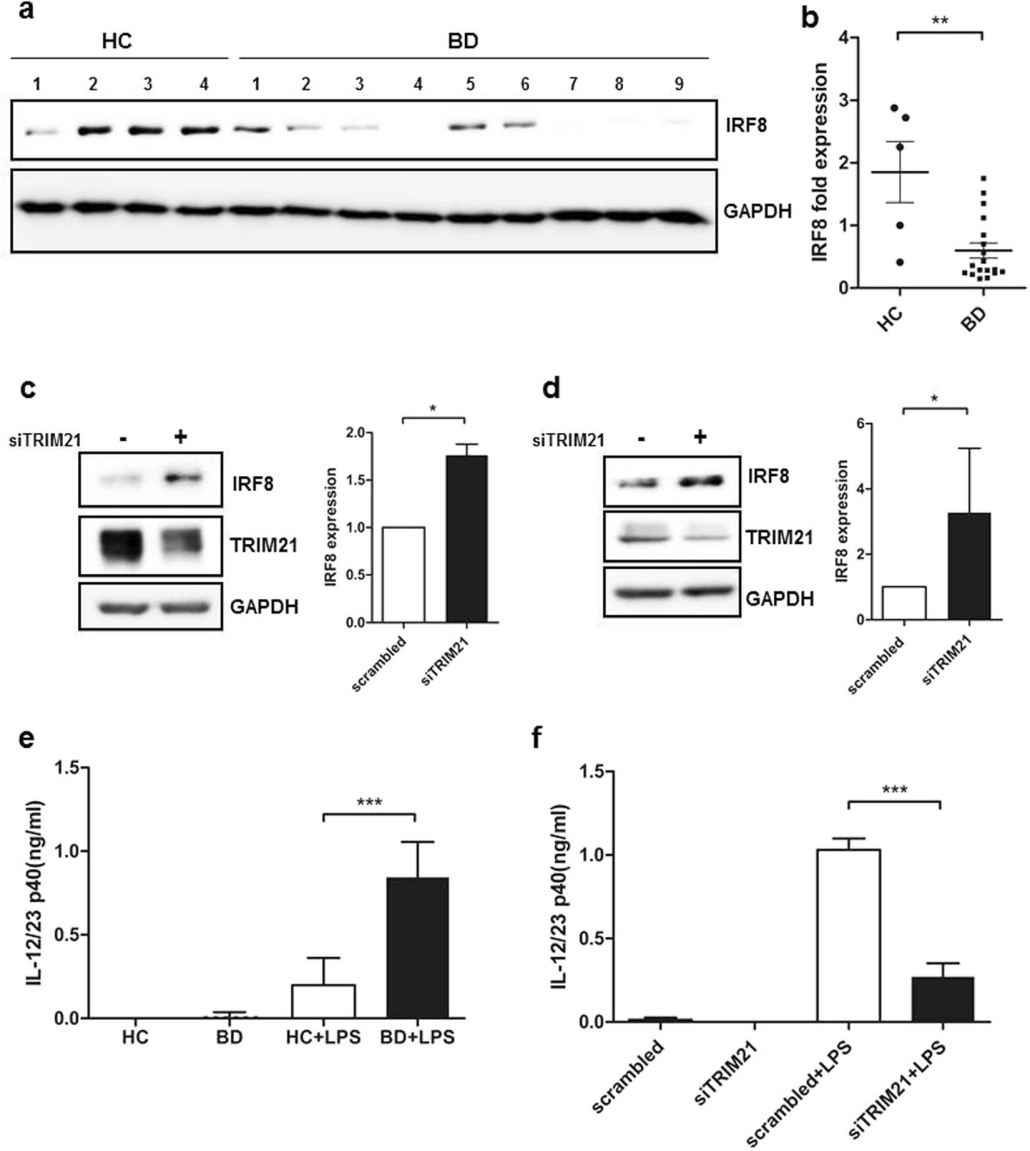
The expression of IRF8, a representative target of TRIM21 ubiquitination, was also decreased in BD monocytes. (**a**) IRF8 protein levels in monocytes from 18 BD patients and five healthy control subjects, as determined by western blot. Cell lysates were from the same sample used in the analysis depicted in Fig. 1a and b. Primary monocytes (**c**) and THP-1 cells (**d**) were transfected with scrambled RNA or TRIM21 siRNA (10 nM) for 24 h. Expression of TRIM21 and IRF8 was detected by immunoblotting. TRIM21 and IRF8 expression was quantitated by densitometry following normalization to GAPDH expression as indicated in the accompanying graph. Data are represented as the mean ± SD. (**e**) Monocytes isolated from healthy controls or BD patients were stimulated with LPS (1 µg/ml) for 24 h. Supernatants were collected, and secretion of IL-12/23p40 was measured by ELISA. (**f**) Primary monocytes from healthy controls were transfected with scrambled RNA or TRIM21 siRNA (10 nM) for 24 h, and the transfected cells were stimulated with LPS (1 µg/ml) for 24 h, and the secretion of IL-12/23p40 was measured by ELISA. Data are represented as the mean ± SD. *P < 0.05. **P < 0.01. ***P < 0.001. The cropped blots are displayed and full-length blots are shown in the Supplementary Information.

TRIM21 interacts with IRF8 upon toll-like receptor(TLR)/IFN-γ stimulation, resulting in TRIM21-mediated ubiquitination of IRF8^16, 17^. IRF8 expression was lower in BD monocytes than in those from healthy controls (Fig. 2a and b). To investigate the direct regulation of IRF8 by TRIM21, TRIM21 was knocked down using siRNA. TRIM21 was silenced using commercially available siRNA duplexes against TRIM21, which resulted in >60% knock-down of TRIM21 in both THP-1 and isolated human monocytes from healthy donors (Supplementary Figure 2A). IRF8 levels increased with TRIM21 knock-down in primary monocytes from healthy controls and THP-1 cells from an established human monocyte cell line (Fig. 2c and d). IRF8 is ubiquitinated by TRIM21, which promotes secretion of IL-12/23p40 after TLR/IFN-γ stimulation^16^. To assess whether IL-12/23p40 secretion was affected by the increased TRIM21, we measured IL-12/23p40 secretion by monocytes from both BD patients and healthy controls after TLR4 stimulation by LPS. BD monocytes exhibited increased secretion of IL-12/23p40 in response to LPS than did healthy monocytes (Fig. 2e). However, BD monocytes with silenced TRIM21 secreted significantly smaller amounts of IL-12/23p40 in response to LPS stimulation (Fig. 2f). From these results, we concluded that TRIM21 down-regulated IRF8 and enhanced the secretion of IL-12/23p40 in BD monocytes.

### BD monocytes induced secretion of Th17 promoting cytokines via TRIM21-dependent pathway

**Figure 3:**
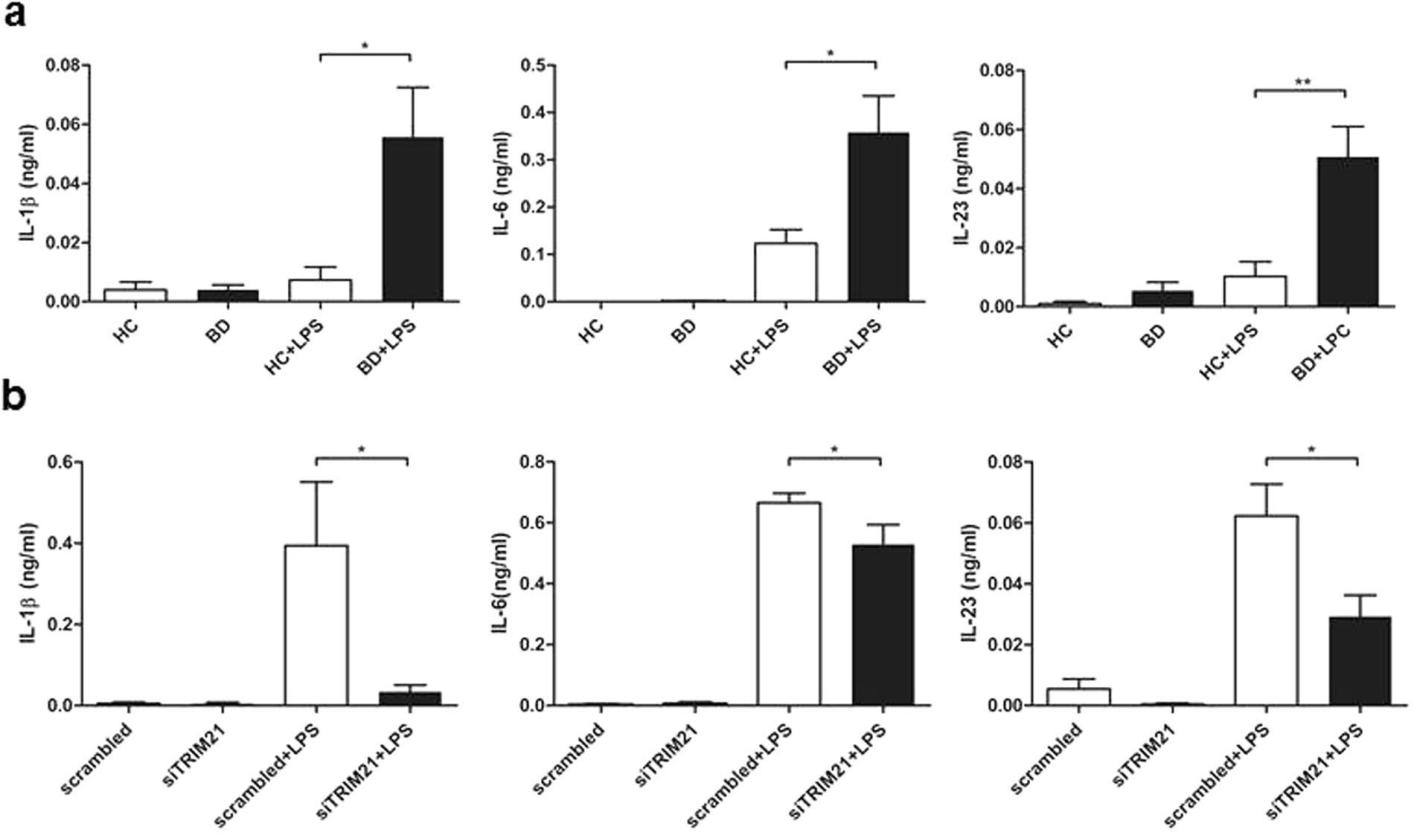
BD monocytes showed increased secretion of Th17-promoting cytokines, and knock-down of TRIM21 decreased cytokine secretion. (**a**) Monocytes isolated from healthy controls or BD patients were stimulated with LPS (1 µg/ml) for 24 h. Supernatants were collected, and secretion of IL-1β, IL-6, and IL-23 was measured by ELISA. (**b**) Primary monocytes from healthy controls were transfected with scrambled RNA or TRIM21 siRNA (10 nM) for 24 h, and the transfected cells were stimulated with LPS (1 µg/ml) for 24 h. Supernatants were collected; and secretion of IL-1β, IL-6, and IL-23 was measured by ELISA. Data are represented as the mean ± SD. *P < 0.05, **P < 0.01 compared with LPS stimulation or TRIM21 knock-down in monocytes in healthy monocytes.

TRIM21 upregulates proinflammatory cytokines through recognized antibody-bound antigen and TLR response. To confirm whether TRIM21 promoted inflammatory cytokine secretion after TLR stimulation, secretion of IL-6, IL-1β, and IL-23 were measured in freshly isolated human monocytes after LPS stimulation. BD monocytes exhibited increased IL-6, IL-1β, and IL-23 secretion compared with healthy control monocytes (Fig. 3a). To identify the role of increased TRIM21 on cytokine secretion in monocytes, we knocked down TRIM21 in freshly separated primary monocytes using siRNA. Knock-down of TRIM21 in monocytes significantly decreased IL-6 and IL-1β cytokine secretion, and IL-23 secretion was also slightly decreased, but not significantly so (Fig. 3b). IL-6, IL-1β and IL-23 are thought to play significant roles in differentiating naïve T cells into pathogenic Th17 cells; our results suggest that increased TRIM21 in monocytes triggered the release of Th17-promoting cytokines under TLR4 stimulation by LPS.

### BD monocytes show increased NF-kB activation after TLR stimulation

**Figure 4:**
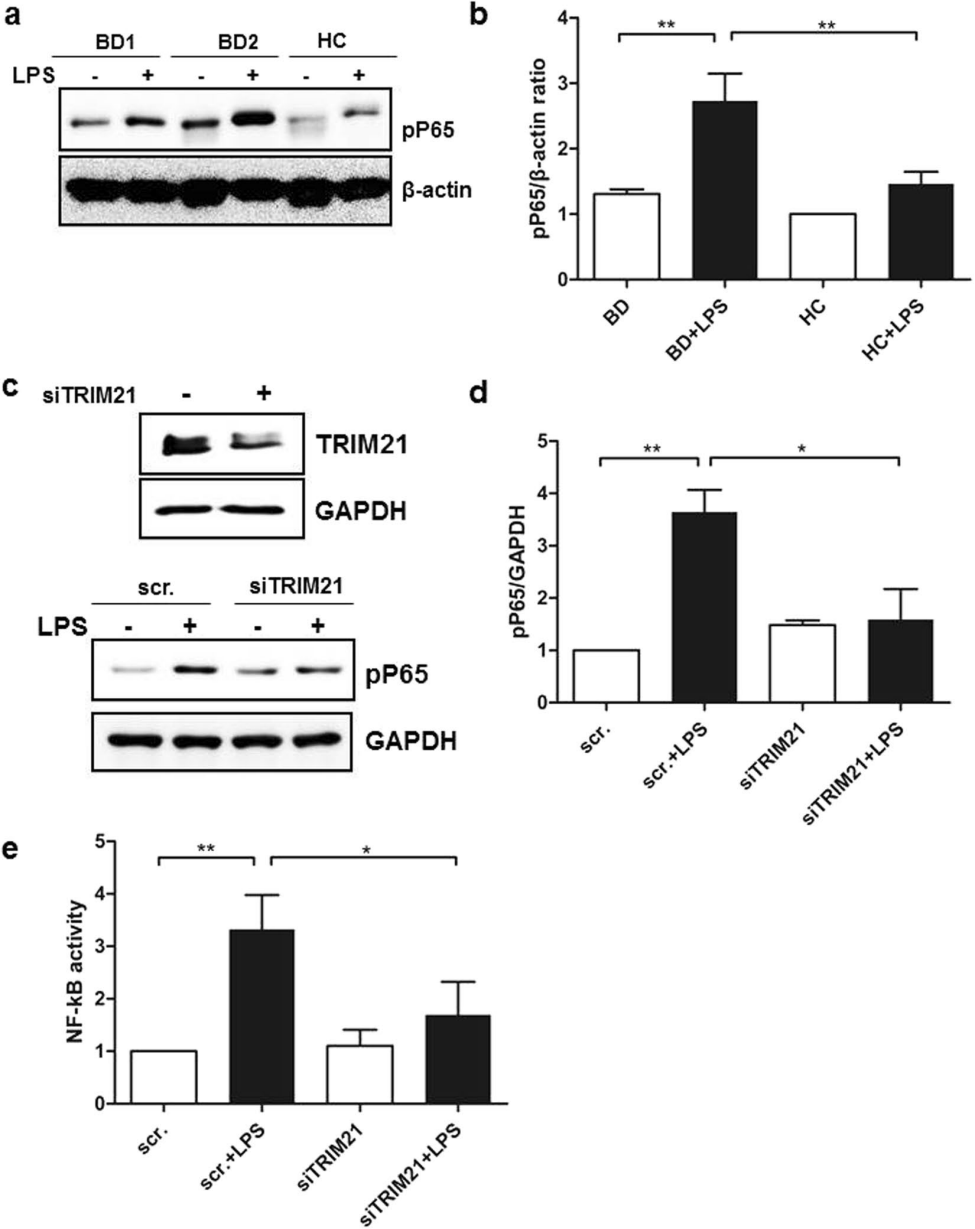
LPS-stimulated BD monocytes exhibited enhanced nuclear translocation of NF-kB. (**a** and **b**) Monocytes isolated from healthy controls and BD patients were stimulated with LPS (1 µg/ml) for 1 h. Cell lysates were evaluated by western blot for levels of phosphorylated p65. β-actin was used as the loading control. A representative result from four independent experiments is shown in (**a**). (**c** and **d**) THP-1 cells were transfected with scrambled RNA or TRIM21 siRNA (10 nM) for 24 h, and the transfected cells were stimulated with LPS (1 µg/ml) for 1 h. (**e**) THP-1 cells were transfected with scrambled RNA or TRIM21 siRNA (10 nM) for 24 h and were incubated with pNF-kB-Luc vector (500 ng) and pβ-galactosidase (500 ng) for 24 h. The transfected cells were stimulated with LPS (1 µg/ml) for 6 h. The results represent NF-kB luciferase activity, normalized for β-galactosidase. Data are represented as the mean ± SD. The cropped blots are displayed and full-length blots are shown in the Supplementary Information.

TRIM21 promotes NF-kB signaling in response to TLR agonists, such as viral and bacterial antigens^1^. We hypothesized that up-regulated TRIM21 might have increased secretion of Th17-promoting cytokines by modulating upstream NF-kB activation. To determine whether the increased TRIM21 enhanced NF-kB activation, monocytes from BD patients and healthy controls were stimulated with 1 µg/ml LPS for 1 h and p-p65 was identified by western blot analysis. BD monocytes exhibited increased p-p65 levels compared with healthy monocytes (Fig. 4a and b), and knock-down of TRIM21 in THP-1 cells using siRNA decreased the level of p-p65 (Fig. 4c and d). To confirm whether TRIM21 regulated the NF-kB pathway, we performed promoter analyses using an NF-kB activity reporter assay in cells stimulated with LPS. Knock-down of TRIM21 in THP-1 cells stimulated with LPS significantly decreased NF-kB reporter activity (Fig. 4e). These results suggest that the enhanced secretion of inflammatory cytokines from BD monocytes may have been mediated by increased NF-kB activation.

### BD monocytes promoted Th17 differentiation in T-cell co-culture

**Figure 5:**
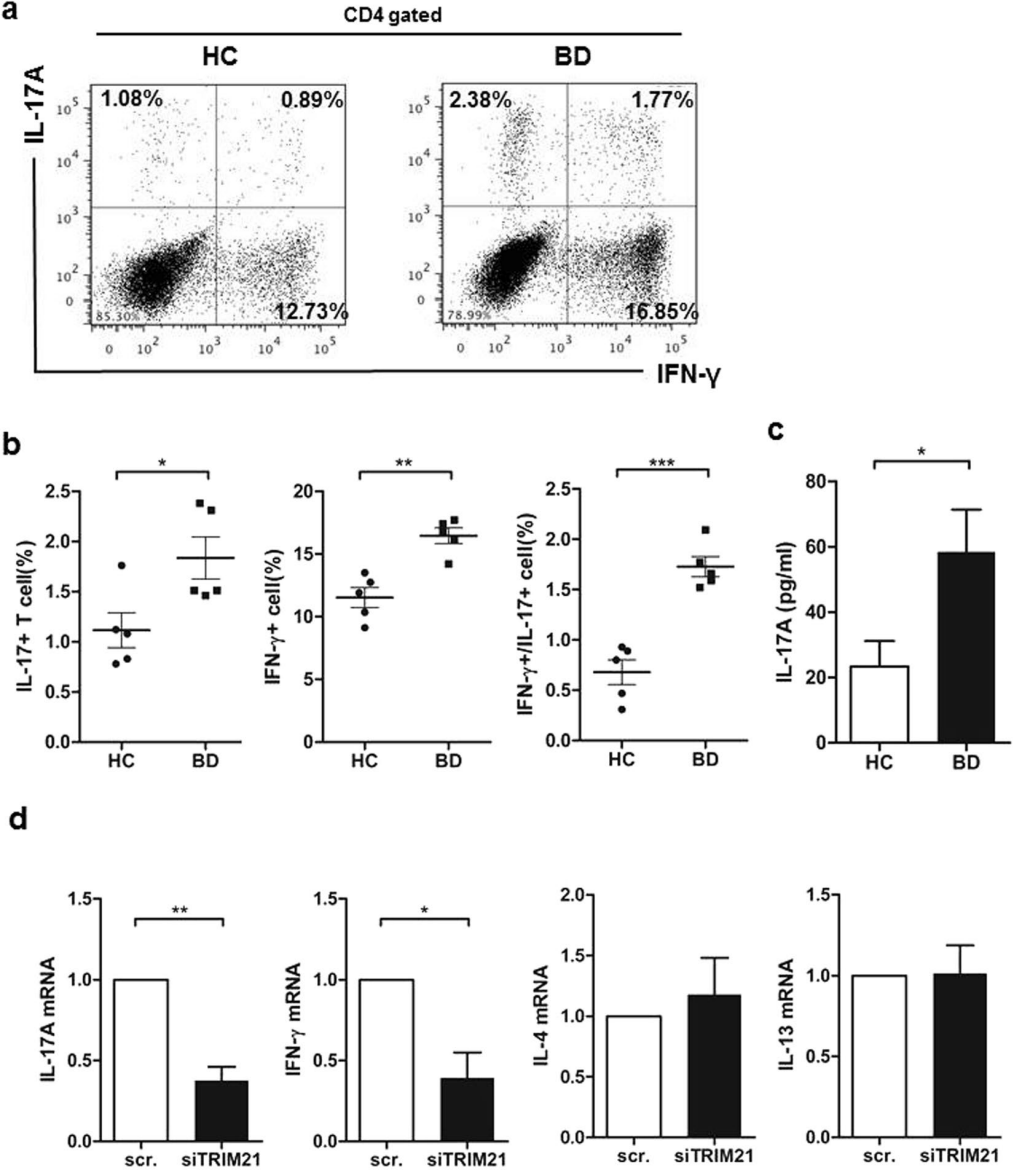
BD monocytes promoted both Th1 and Th17 polarization after co-culture with effector T cells. (**a** and **b**) Primary monocytes from healthy controls or BD patients were co-cultured with allogeneic naïve CD4 + T cells in the presence of anti-CD3 (1 µg/ml) mAb and LPS (100 ng/ml) for 7 days. Cells were re-stimulated with PMA (50 ng/ml) and ionomycin (1 µg/ml) for 6 h in the presence of GolgiPlug for 4 h, and then expression of IL-17 and IFN-γ were determined by intracellular flow cytometry. (**c**) Production of IL-17A in monocyte/T-cell co-culture supernatant was quantified by ELISA. Data are represented as the mean ± SD. **P < 0.01. (**d**) Knock-down TRIM21 in monocytes was co-cultured with allogeneic naïve CD4 + T cells in the presence of anti-CD3 (1 µg/ml) mAb and LPS (100 ng/ml) for 48 h. Total RNA was extracted and analyzed by RT-PCR for mRNA expression of *IL17A*, *IFNG*, *IL4*, and *IL13*. Data are represented as the mean ± SD.

To investigate the pathophysiologic significance of cytokines produced by BD monocytes, we performed co-cultures with BD monocytes and responder CD4 T cells as previously described^18^. After 7-day co-culture using monocytes (4 × 10^5^/ml) and allogeneic responder CD4^+^ T cells (4 × 10^5^/ml) from healthy controls, the proportion of IL-17^+^ and IL-17^+^/IFN-γ^+^ producing T cells was increased in BD monocyte-responder T cell cultures compared with that in control monocyte-responder T cell co-cultures (Fig. 5a and b). The increased soluble IL-17A production in BD monocyte- responder T cell co-culture supernatant was confirmed using ELISA (Fig. 5c). Interestingly, responder CD4 + T-cell culture with TRIM21-silenced BD monocytes significantly lowered mRNA expression of both *IL17A* and *IFNG*, but not *IL4* or *IL13*, using Th2 cytokines as control (Fig. 5d). Collectively, these results support the conclusion that the Th1- and Th17-priming role of BD monocytes was partly dependent on increased TRIM21.

## Discussion

We have uncovered a novel regulatory function of TRIM21, one that plays a critical role in inflammatory cytokine production in activated monocytes from BD patients. TRIM21 regulated important target pathways, such as IRF8 and NF-kB, and modulated proinflammatory cytokine production in monocytes.

The evidence supports a new pathogenic model in which the modulation of ubiquitination is responsible for the distinct immune reaction in BD^19^. TRIM proteins activate innate immune signaling pathways, including NF-kB, AP-1, and IRFs, by generating ubiquitin signals^20^. A recent genetic study on BD revealed that TRIM39 is associated with a genetic predisposition to this disease^21^. TRIM39 regulates the NF-κB signal negatively by modulating the production of inflammatory cytokines, such as TNF-α^19^. From the classical view on the immunologic abnormalities in BD patients, strong Th1 immune response occurs in active BD^22^. BD patients exhibit high levels of TNF-α, IFN-γ and IL-1 in peripheral circulation^10, 23^. This recent research on TRIM39 revealed TRIM protein modulation of ubiquitination as important in skewed immune polarization in BD. In the future, we hope to discover the role of TRIM21 protein in the fine-tuning of immune polarization, especially in Th17 response.

TRIM21 interacts with numerous proteins involved in both innate and adaptive immunity^24^. The primary role of TRIM21 is to sense intracellular antibodies during viral infection, but TRIM21 also activates innate immune signaling pathways, including NF-kB and IRFs^20^. Recent linkage analysis found IRB8, an ubiquitinating target of TRIM21, is altered in BD; and dysregulation of IRF8 is associated with altered cytokine expression, especially Th17 response in BD^25, 26^. IRF8 regulates the expression of inflammatory cytokines, such as IL-12/23p40, in macrophages and, presumably, their precursors, monocytes^16^. Active BD exhibits increased Th17 response characterized by increased IL-17, which prompts us to consider the pathogenic role of this proinflammatory pattern in the pathogenesis of BD^9, 10, 25^. Consistent with our results from the co-cultures between monocyte and responder T cells, researchers have observed excessive CD4^+^ T cells producing IL-17 and IFN-γ (Th1/Th17) cells in BD patients^27^.

Th17 cells are characterized by type 17 signature cytokines, such as IL‑17, IL‑22, IFN-γ, and GM‑CSF. The development and expansion of Th17 cells is dependent on specific cytokine combinations, such as IL‑1β, IL‑6, and TGF-β; and IL-23 stabilizes and potentiates the pathogenic ability of Th17^28^. The main cellular sources of these Th17-promoting cytokines varies depending on the cytokines, but monocytes are also an important source of IL‑1β or IL‑6^29^. We demonstrated that repressed TRIM21 expression in monocytes inhibited Th17 and Th1 immune deviation, not Th2 response, using siRNA and the co-culture method. TRIM21 regulated the production of IL‑1β and IL‑6 from LPS-activated monocytes. Although IL-23 induces Th17 response during T-cell development, IL-23 is not indispensable in priming the Th17 cells^28^.

TRIM21 directly regulates important signaling-modulated inflammation, including IRF and NF-kB pathway, as mentioned earlier^20^. The regulation of IRF8 by TRIM21 is suggested directly via ubiquitination in the nucleus of macrophages^16^. Again, from our data, when we suppressed the expression of TRIM21 in both primary monocytes and THP-1 cells, IRF8 was significantly down-regulated. IRF8 regulates the differentiation and functions of monocytes^30^. Moreover, IRF8 regulates macrophage maturation, survival, and innate immune responses, including autophagy^31^. In addition, IRF8 induces a cytokine milieu that favors Th1 and Th17 response by enhancing TGF-β production from antigen-presenting cells^32^. However, the regulation of the NF-kB pathway by TRIM21 appears to be more complicated. Although TRIM21 is not required for constitutive NF-κB signaling, TRIM21 activates NF-κB signaling after intracellular sensing of pathogen^1^. The stimulation of TRIM21 on NF-κB activation is dependent on various signaling, such as Transforming growth factor beta-activated kinase 1 (TAK1), IκB kinase-α (IKKα), and IκB^1^. Interestingly, TRIM21-/- mice showed increased dysregulation of NF-kB-mediated proinflammatory cytokine production^33, 34^. Because TRIM21 regulates various signaling pathways on different cell types *in vivo*, overall biological consequences may demonstrate conflicting results from various cellular levels.

The main limitation of our study is that we did not clearly define the role of monocyte in co-culture with responder T cells. Monocytes play key roles during inflammation and pathogen challenge. Different subsets of monocytes exhibit different functions, including coordination of inflammation by recruiting inflammatory cells and generating monocyte-derived dendritic cells or macrophages^35^. Different monocyte subsets have been identified by differential expression of CD14 and CD16^36, 37^. Classical monocytes, which are strongly positive for CD14, constitute the majority of monocytes in healthy individuals; and monocytes expressing CD16 account for less than 15% of all monocytes during homeostasis but increase significantly during infectious diseases and inflammatory disorders^38, 39^. BD patients also demonstrated increased CD16+ monocytes in peripheral blood (our observation, unpublished). These “non-classical” CD16+ monocytes are the primary producers of inflammatory cytokines IL-1β and TNF-α in inflammatory conditions^40^. Interestingly, recent data suggests TRIM21 can be the factor that activates the proinflammatory properties of CD16+ monocytes^41^. In this context, certain monocyte subsets will be associated with increased TRIM21 expression and the proinflammatory condition in BD. Thus, TRIM21 expression in different monocyte subsets should be investigated in futures studies.

TRIM proteins act as viral sensors and antiviral molecules^1, 15^. The role of viral infections, such as herpes simplex virus infection, in the BD pathogenesis has long been investigated^42^. Although the fundamental role of TRIM21 in herpesviruses, double-stranded DNA viruses, has not been reported; but the TRIM protein modulation of ubiquitination appears to be instrumental in the link between environmental factor and immunological disease.

In summary, our study revealed a previously unrecognized role of TRIM21 in regulating the secretion of proinflammatory cytokines from monocytes in BD. We found that TRIM21 was elevated and IRF-8 was down-regulated in monocytes from BD. Also, TRIM21 in monocytes stimulated NF-kB signaling in BD monocyte. Finally, monocyte-derived proinflammatory cytokines, which promote Th1/Th17 inflammation, were regulated by TRIM21. Our results may have important implications for understanding the pathogenic role of the innate immune system in chronic inflammatory conditions.

## Electronic supplementary material

Supplement Figures and Information
