# Supplementary material for: Enhancement of Th1/Th17 inflammation by TRIM21 in Behçet’s disease
Source: Sci Rep. 2017 Jun 7;7:3018. doi: 10.1038/s41598-017-03251-5 (PMC5462739; doi:10.1038/s41598-017-03251-5)
Supplement: Supplementary file 1 — Supplement Figures and Information [file 41598_2017_3251_MOESM1_ESM.pdf]

SUPPLEMENT INFORMATION

Enhancement of Th1/Th17 inflammation by TRIM21 in Behçet's disease

Yuri Ahn, MS[1], Ji-Hye Hwang, MS[1], Zhenlong Zheng, MD, PhD[1,2], Dongsik Bang, MD, PhD[3], and Do-Young Kim, MD[1*]

[1]*Department of Dermatology and Cutaneous Biology Research Institute, Yonsei University College of Medicine, Seoul, Korea*

[2]*Department of Dermatology, Yanbian University Hospital, Yanji, China*

[3]*Department of Dermatology, Catholic Kwandong University, International St. Mary's Hospital, Incheon, Korea*

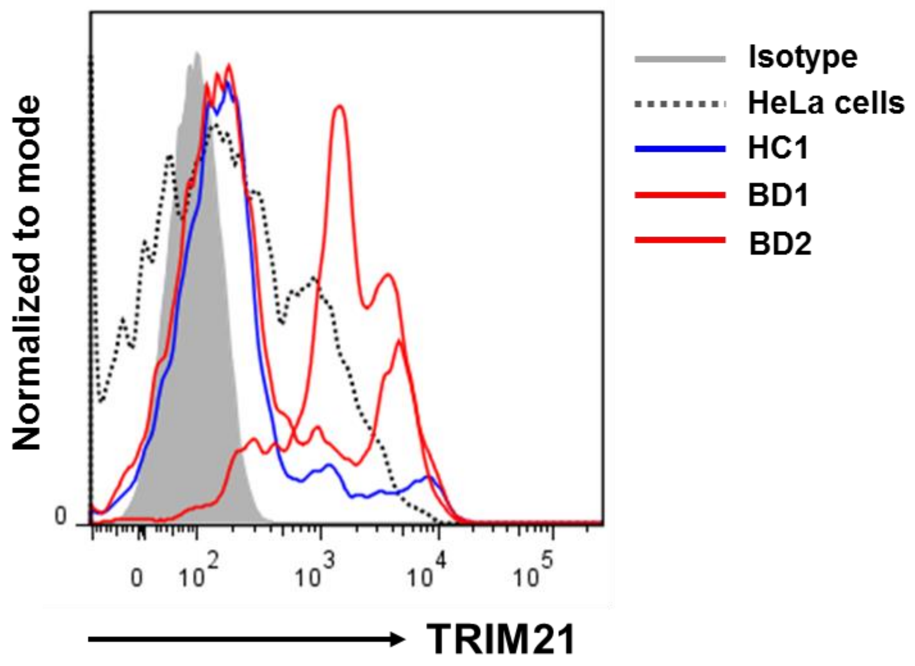

Supplementary Figure 1. TRIM21 expression in monocytes were determined by flow cytometry using the anti-TRIM21 mAb and the IgG1 control isotype mAb(gray histogram). TRIM21 expression in BD monocytes (red line) was increased compared with monocyte from healthy control (blue line). HeLa cells (dotted line), which have elevated TRIM21 levels, were used as positive control. Results are representative from three independent experiments.

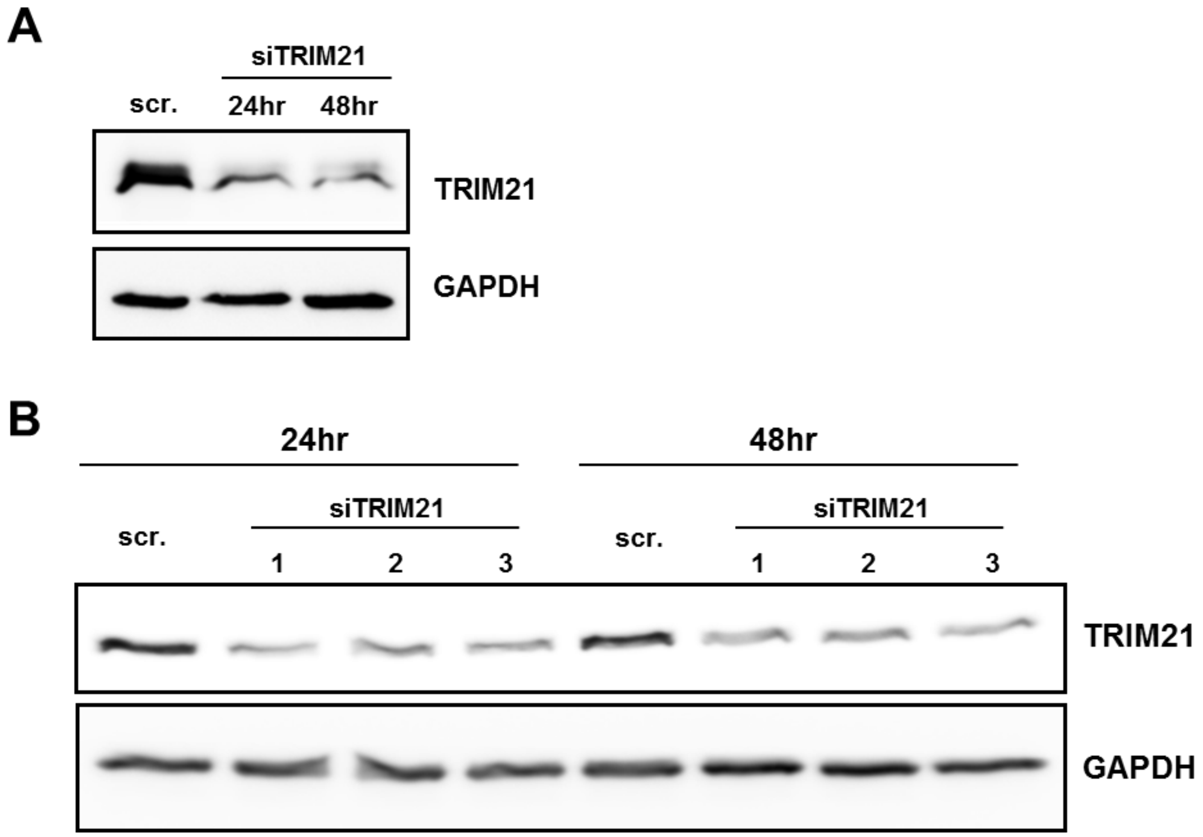

Supplementary Figure 2. The knockdown efficiency of TRIM21 in primary monocytes and THP-1 cells. A and B, Immunoblot analysis of the knockdown of endogenous TRIM21 in primary monocyte and THP-1 cells were transfected with scrambled RNA or TRIM21 siRNA for 24 hr and 48 hr. GAPDH serves as a loading control.

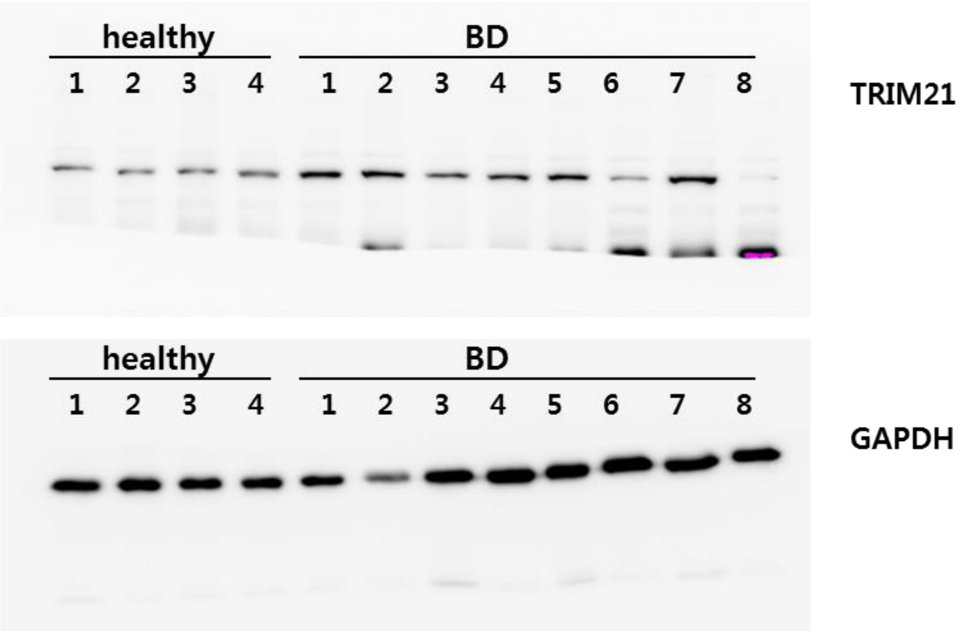

Supplementary Information for Figure 1a. Full-length gels and blots for the cropped blots in Figure 1a

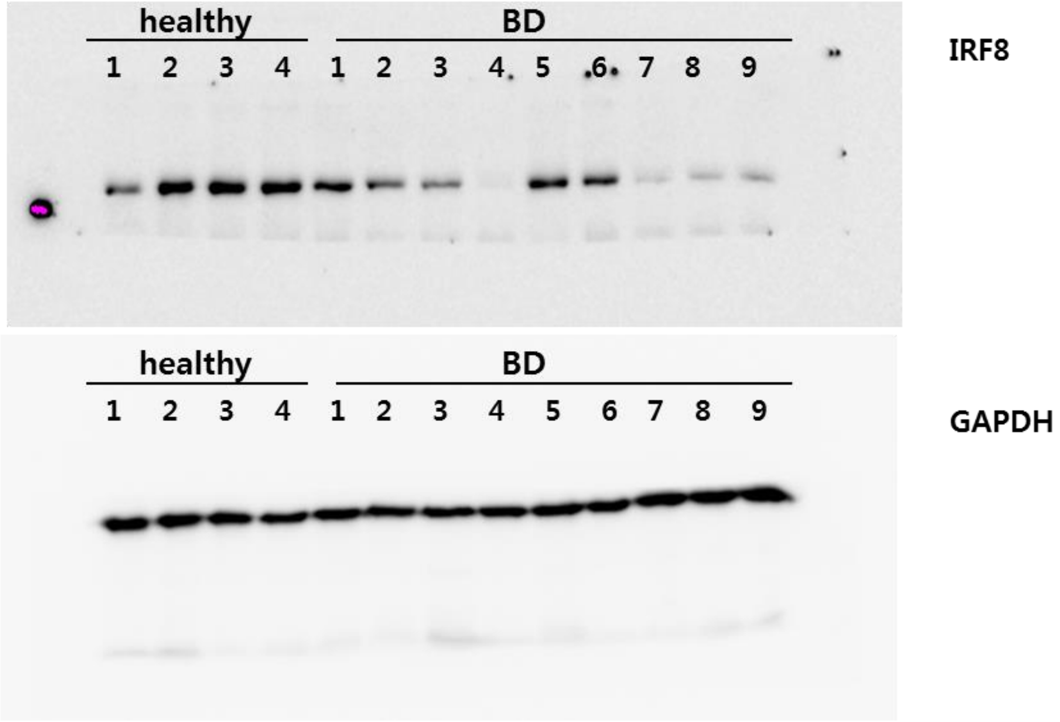

Supplementary Information for Figure 2a. Full-length gels and blots for the cropped blots in Figure 2a

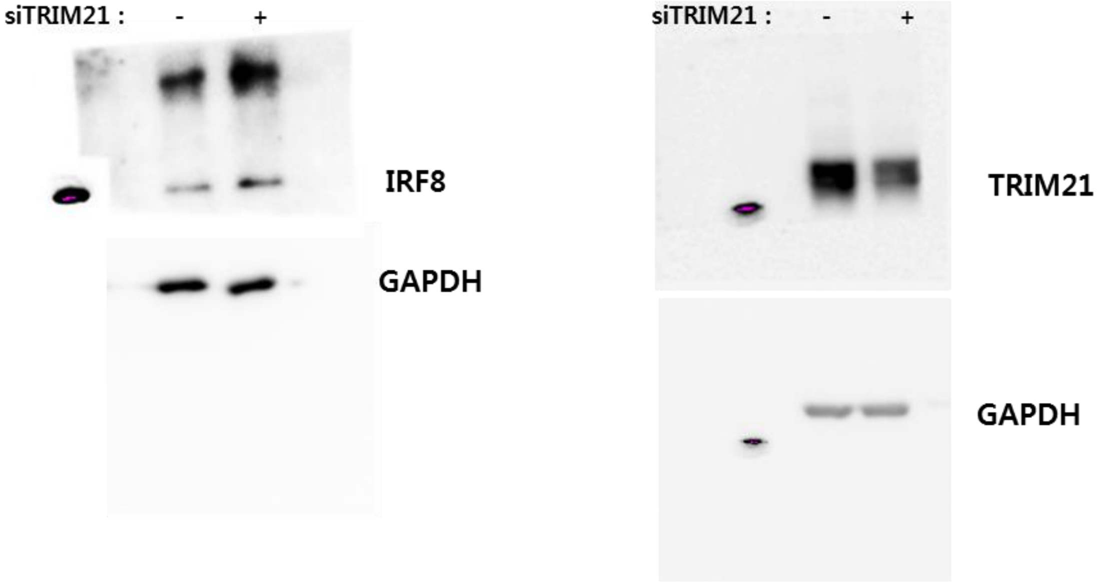

Supplementary Information for Figure 2c. Full-length gels and blots for the cropped blots in Figure 2c

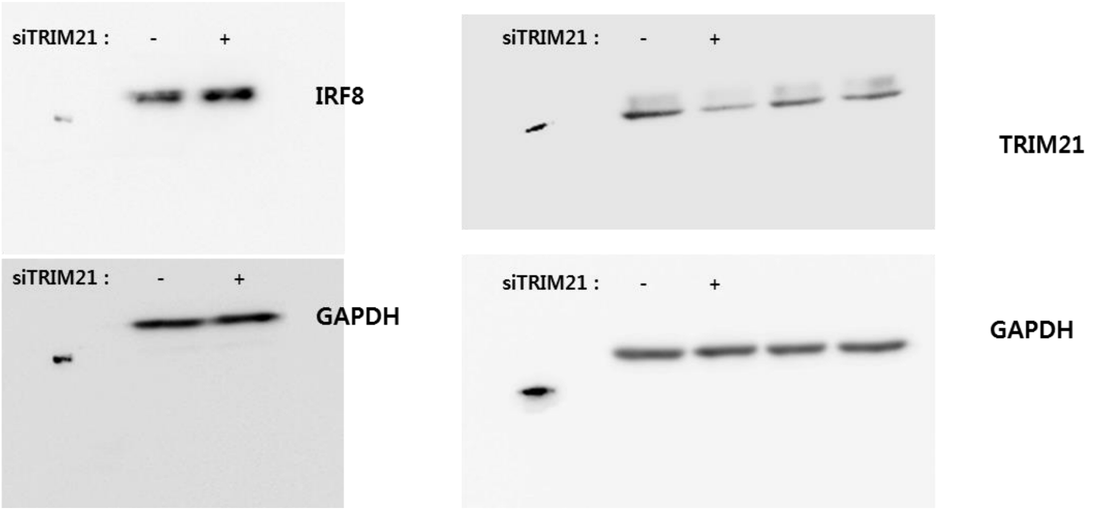

Supplementary Information for Figure 2d. Full-length gels and blots for the cropped blots in Figure 2d

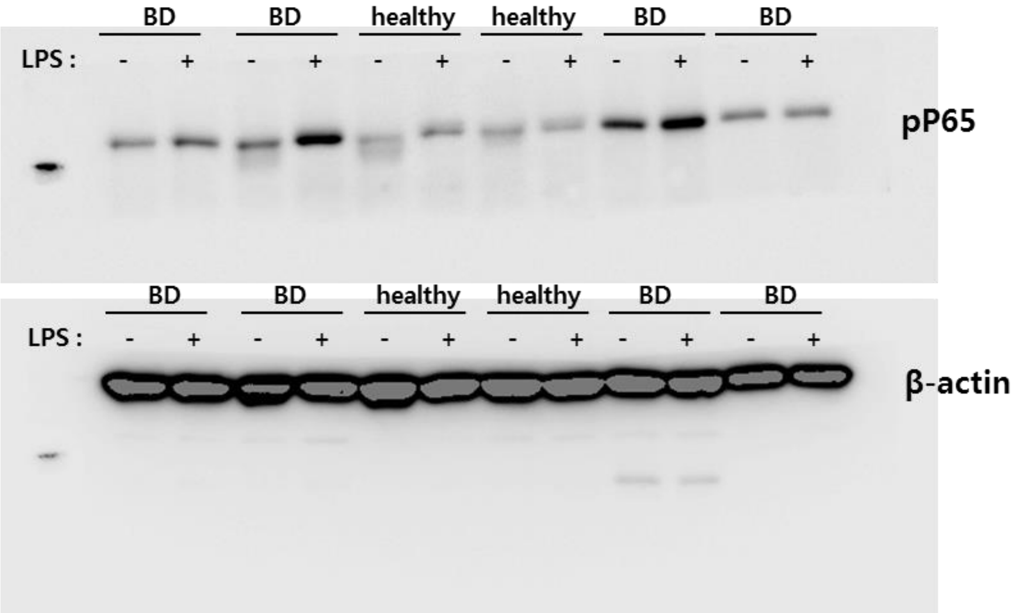

Supplementary Information for Figure 4a. Full-length gels and blots for the cropped blots in Figure 4a

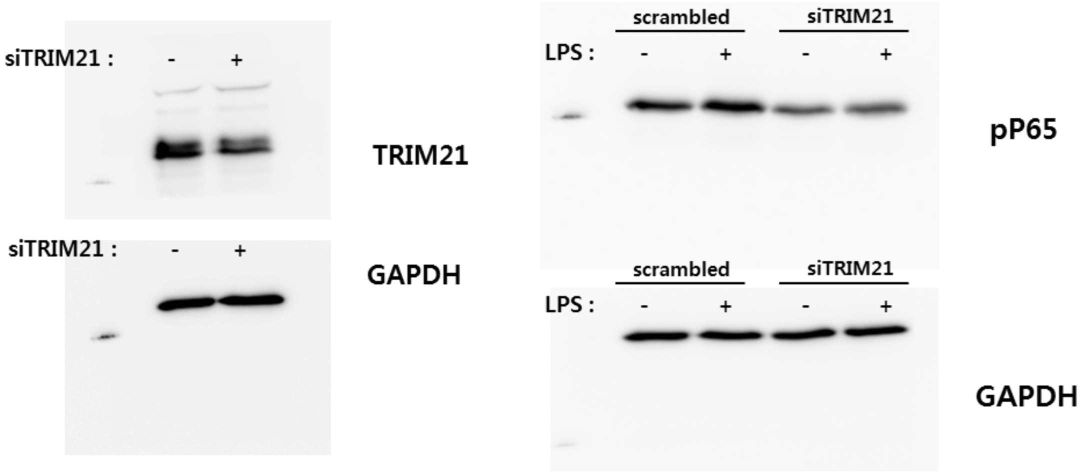

Supplementary Information for Figure 4c. Full-length gels and blots for the cropped blots in Figure 4c
